# Safety evaluation of Sodium-glucose cotransporter 2 inhibitors for cancer risk in specific populations: systematic review and meta-analysis

**DOI:** 10.3389/fcdhc.2026.1775359

**Published:** 2026-05-08

**Authors:** Jing Teng, Tai Li, Ying Tan, Huifang Tang

**Affiliations:** 1 Institute of Cardiovascular Disease, Hengyang Medical School, The First Affiliated Hospital, University of South China, Hengyang, Hunan, China; 2 Department of Cardiology, The First Affiliated Hospital, Hengyang Medical School, University of South China, Hengyang, Hunan, China; 3 Hunan Provincial Key Laboratory of Multi-Omics and Artificial Intelligence of Cardiovascular Diseases, University of South China, Hengyang, Hunan, China

**Keywords:** cancer risk, chronic kidney disease, heart failure, meta-analysis, SGLT 2 inhibitors, type 2 diabetes mellitus

## Abstract

**Aims:**

Sodium-glucose cotransporter 2 (SGLT2) inhibitors, commonly treated for Type 2 diabetes mellitus (T2DM), have demonstrated benefits in reducing cardiovascular and renal events. However, heart failure (HF) and chronic kidney disease (CKD) are prevalent comorbidities in T2DM. This study aims to assess the long-term safety of SGLT2 inhibitor, particularly concerning cancer risk in these populations.

**Methods:**

We searched PubMed, CENTRAL, Web of Science, and ClinicalTrials.gov for studies published up to April 16, 2024. The primary outcome was overall cancer risk, with secondary outcomes focused on specific cancer types. Pooled risk ratios (RR) with 95% confidence intervals (CI) were calculated. This meta-analysis was registered in PROSPERO (CRD42024560310).

**Results:**

28 randomized controlled trials involving 98,297 participants were included. SGLT2 inhibitors did not increase overall cancer risk (RR = 1.05, 95% CI [0.99, 1.12]). Furthermore, the use of SGLT2 inhibitors does not exhibit a significant association with the risk of several common cancer types. Subgroup analyzes indicated that SGLT2 inhibitors appeared to be temporarily safe in patients with HF (RR = 1.03, 95% CI [0.91, 1.18]), CKD (RR = 1.02, 95% CI [0.90, 1.16]) and T2DM related comorbidities. Additionally, none of the different types of SGLT2 inhibitors increase the overall cancer risk.

**Conclusions:**

SGLT2 inhibitors do not appear to increase overall cancer risk, including several common cancers such as breast cancer and bladder cancer. The use of SGLT2 inhibitors in different patient populations is temporarily safe.

**Systematic review registration:**

https://www.crd.york.ac.uk/prospero/, identifier CRD42024560310.

## 1 Introduction

Sodium-glucose cotransporter 2 (SGLT2) inhibitors are a class of medications that have transformed the management of type 2 diabetes mellitus (T2DM) in recent years. SGLT2, a protein located in the proximal tubules of the kidney, is responsible for reabsorbing about 90% of the glucose. Inhibition of SGLT2 increases glucose excretion, thereby reducing blood glucose levels (1–6). Due to their broader benefits, SGLT2 inhibitors are a cornerstone in T2DM treatment, endorsed by both the American Diabetes Association (ADA) and the European Association for the Study of Diabetes (EASD) (7, 8). Beyond glucose control, SGLT2 inhibitors significantly reduce the risk of major adverse cardiovascular events (MACE) (9–11), including cardiovascular death, myocardial infarction, and stroke, as shown in trials like EMPA-REG OUTCOME, where Empagliflozin reduced MACE risk by 14% (12). They also lower the risk of heart failure-related hospitalizations, demonstrated by a 26% reduction in the DAPA-HF trial with Dapagliflozin in heart failure (HF) with reduced ejection fraction (13). Additionally, SGLT2 inhibitors slow the progression of chronic kidney disease (CKD) in T2DM patients. In the CREDENCE trial, Canagliflozin was shown to reduce the risk of progression to end-stage kidney disease and renal-related death by over 30% (14). These outcomes highlight the multifaceted benefits of SGLT2 inhibitors, extending beyond glycemic control to include substantial cardiovascular and renal health protection, which are critical complications of T2DM (15, 16).

Given the widespread use of SGLT2 inhibitors in patients with T2DM, HF, and CKD populations, there is increasing attention to their long-term safety, particularly in relation to cancer risk. While their therapeutic efficacy is well-established, potential adverse effects remain a concern. Preclinical studies suggest that SGLT2 inhibition may modulate cancer cell growth by altering glucose metabolism, insulin signaling, and cellular proliferation (17–19). Reduced glucose may deprive proliferating tumor cells of an essential energy source, potentially inhibiting tumor growth (20–23). *In vitro* studies show that SGLT2 inhibitors can reduce glucose uptake in certain cancer cells, thereby impairing cell proliferation (24–26). However, glucose deprivation may drive tumor cells to utilize alternative metabolic pathways that could promote tumor survival and progression (27–31).

Although some previous studies have explored the relationship of SGLT2 inhibitors and cancer incidence, the findings remain inconclusive (32, 33). For example, a meta-analysis of 46 trials (involving 34,569 participants) suggested an increased risk of bladder cancer (34), while another meta-analysis of 27 trials (involving 48,185 participants) found no significant association (35). Despite the clinical endorsement of SGLT2 inhibitors as first-line agents for T2DM, HF, and CKD, owing to their demonstrated cardiovascular and renal benefits, the impact of SGLT2 inhibitors on cancer risk in these populations has not been systematically assessed (36, 37).

Most pivotal trials on SGLT2 inhibitors have follow-up durations of less than five years, and cancer-related outcomes are relatively rare events. In real-world clinical practice, patients with T2DM, HF, and CKD often involves complex interactions of various risk factors, such as age, lifestyle, and genetics. Thus, a comprehensive meta-analysis focusing on the long-term safety of SGLT2 inhibitors, particularly cancer risk, is essential for clearer insights. This study aims to address this gap by systematically reviewing data from randomized controlled trials to evaluate cancer risk associated with SGLT2 inhibitors across diverse patient populations and provide updated evidence to inform clinical decision-making.

## 2 Methods

This meta-analysis was conducted in accordance with the 2020 Preferred Reporting Items for Systematic Reviews and Meta-Analyzes (PRISMA) statement (38) and was registered in PROSPERO (CRD42024560310). The findings were reported following the Grading of Recommendations, Assessment, Development, and Evaluations (GRADE) guidelines (39).

### 2.1 Search strategy and study selection

A comprehensive search was conducted in PubMed, the Cochrane Central Register of Controlled Trials (CENTRAL), Web of Science, and ClinicalTrials.gov from inception to April 16, 2024, using relevant search terms, with no restrictions on language, publication date, or article type. Additionally, references from included studies, reviews, and meta-analyzes were examined to identify further relevant published and unpublished trials on ClinicalTrials.gov. The detailed search strategy is presented in **Supplementary Table 1**.

Two investigators independently screened studies for inclusion. Trials were included according to following criteria (1): published or unpublished randomized controlled trials (2); adults with underlying medical conditions, including T2DM, HF, and CKD (3); treatment with SGLT-2 inhibitors (as a single agent) for the experimental group, with a placebo for the control group (4); trials with a minimum of 12 weeks of follow-up; and (5) studies reporting any cancer outcomes.

Cancer events were recorded as serious adverse events and were identified using pre-specified lists from the Medical Dictionary for Regulatory Activities (MedDRA). Meeting abstracts were excluded due to insufficient information on trial characteristics, outcome definitions, and quality assessments.

### 2.2 Data extraction

Published full-text articles were used as the primary data sources, retrieve the NCT numbers registered with published research institutes to ClinicalTrials.gov and extract data. Data extraction was performed independently by two researchers. The extracted data included (1): study characteristics, such as authors, year of publication, sample size, and duration of follow-up (2); participant characteristics, including mean age, sex ratio, and underlying diseases (3); the incidence of total and site-specific cancers, including primary, recurrent, and metastatic cancer, as well as different cancer types (4); the drug dosages and administered to the SGLT2 inhibitors group. We extracted the final number of cancer patients as the outcome variable for analysis, which was a binary variable.

For the three types of cancer under review, bladder cancer, prostate cancer, and breast cancer, the final number of cancer patients included in the analysis consisted only of those who were confirmed to have cancer. Tumors such as papillomas or benign tumors were not included in the final count or analysis. This ensured that only confirmed cancer diagnoses were considered in the results.

### 2.3 Quality assessment

The risk of bias of included randomized controlled trials (RCTs) was assessed using the Cochrane Risk of Bias Tool. Studies were classified as having “low risk,” “high risk,” or “unclear risk” risk of bias based on the following domains: random sequence generation, allocation concealment, blinding, completeness of outcome data, selective reporting of outcomes, and other biases. Discrepancies were resolved through discussion or adjudication by a third reviewer.

### 2.4 Outcomes of interest

The primary outcome was the overall incidence of cancer. Subgroup analyzes were performed based on (1): cancer types (2), T2DM, HF, CKD, and T2DM comorbidities, and (3) types of SGLT2 inhibitors.

### 2.5 Statistical analysis

Risk ratios (RRs) with 95% confidence intervals (CIs) were calculated using the Mantel–Haenszel method. Heterogeneity was assessed with the I² statistic; a fixed-effects model was applied when I² < 50%, otherwise a random-effects model was used. Given the low heterogeneity observed, a fixed-effects model was adopted.

Publication bias was assessed using funnel plots, Egger’s and Begg’s tests. All analyzes were conducted using RevMan 5.3, and R Studio 4.3.0.

## 3 Results

### 3.1 Study selection and characteristics

**Figure 1:**
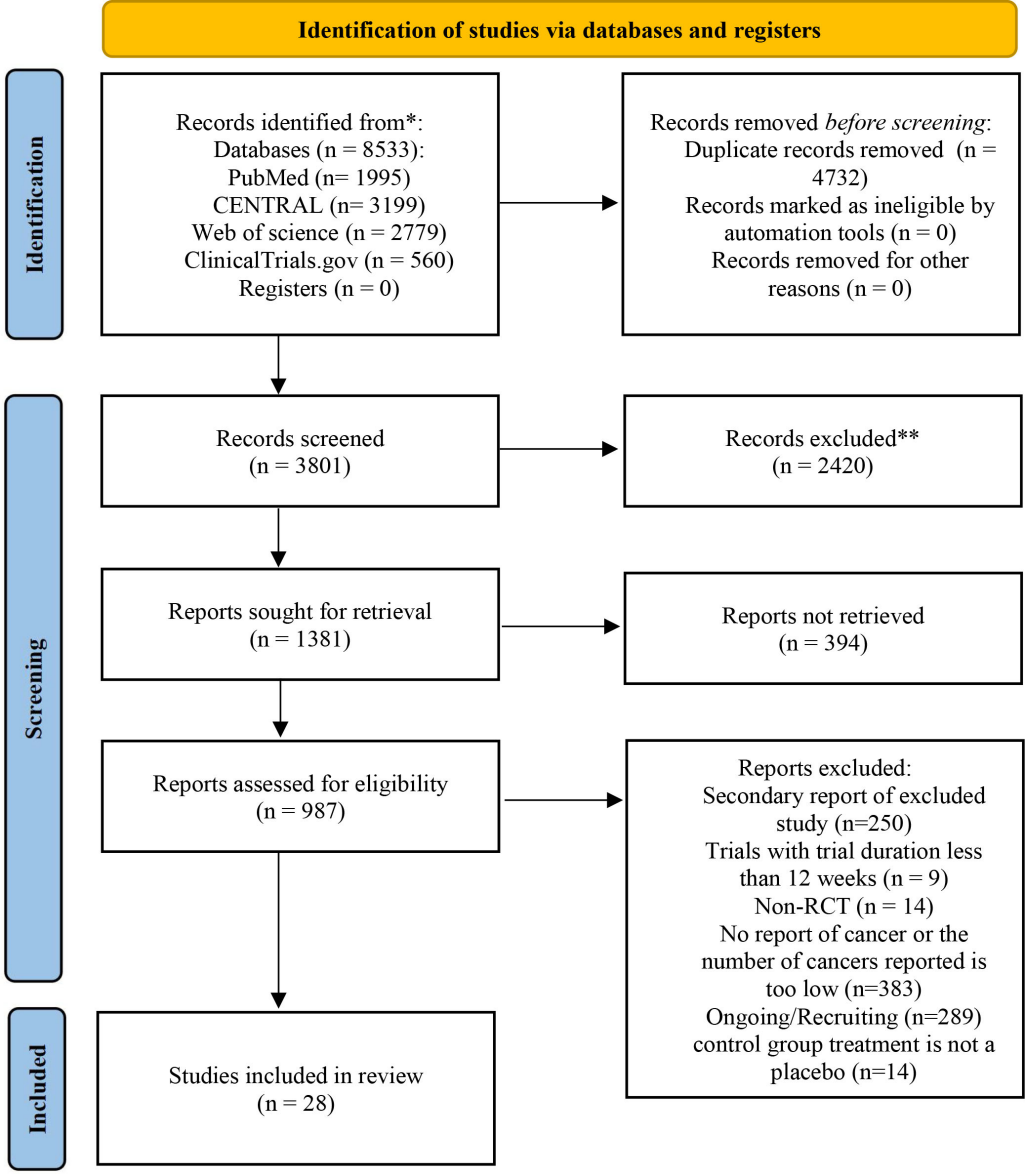
PRISMA diagram of study selection in the meta-analysis.

A total of 8,533 records were identified, of which 28 RCTs involving 98,297 participants met the inclusion criteria (**Figure 1**).

The 28 trials involved 98,297 participants, of whom 53,255 were assigned to the SGLT2 inhibitor intervention group (including Ertugliflozin, Bexagliflozin, Dapagliflozin, Empagliflozin, Canagliflozin, and Sotagliflozin), and 45,042 participants were assigned to the control group (placebo). The mean age of the participants ranged from 59.3 to 71.9 years, and the proportion of male participants ranged from 45.5% to 76.6%. The sample sizes of individual trials ranged from 272 to 17,143, with an average follow-up duration of 124.2 weeks (range: 13 to 338 weeks). The breakdown of studies for each SGLT2 inhibitor included: three trials for Ertugliflozin, one for Bexagliflozin, eight for Dapagliflozin, eight for Empagliflozin, three for Canagliflozin, and five for Sotagliflozin (**Table 1**).

**Table 1:** Overall baseline characteristics of the studies included in the meta-analysis.

First author	Year	Number of patients	Follow-up time	Mean age (standard deviation)	Sex		Target population	Therapeutic regimen	
					Male	Female		Experiment	Control
Wilding (40)	2012	807	104Weeks	59.3 (8.22)	382	425	T2DM	Dapagliflozin2.5/5/10 mg	Placebo
Boehringer (41)	2014	494	78Weeks	58.8 (9.9)	276	218	T2DM	Empagliflozin10/25 mg	Placebo
Dagogo-Jack (42)	2018	462	52Weeks	59.1 (9.0)	263	199	T2DM	Ertugliflozin5/10/15 mg	Placebo
AstraZeneca (43)	2017	272	24Weeks	57.5 (8.70)	130	142	T2DM	Dapagliflozin10 mg	Placebo
Wason (44)	2021	375	110Weeks	66.3 (6.5)	209	166	T2DM	Sotagliflozin200/400 mg	Placebo
Rosenstock (45)	2014	563	52Weeks	56.7 (9.5)	256	307	T2DM	Empagliflozin10/25 mg	Placebo
McMurray (13)	2019	4736	111Weeks	66.3 (10.9)	3635	1101	HFrEF	Dapagliflozin10 mg	Placebo
Packer (46)	2020	3726	148Weeks	66.8 (11.0)	2837	889	HFrEF	Empagliflozin10 mg	Placebo
Anker (47)	2021	5985	200Weeks	71.9 (9.4)	3312	2673	HFpEF	Empagliflozin10 mg	Placebo
Solomon (48)	2022	6253	168Weeks	71.7 (9.6)	3516	2737	HFpEF	Dapagliflozin10 mg	Placebo
Voors (49)	2022	524	13Weeks	68.5 (13.2)	351	173	Acute HF	Empagliflozin10 mg	Placebo
Wheeler (50)	2020	4298	153Weeks	61.8 (12.1)	2879	1419	CKD	Dapagliflozin10 mg	Placebo
Herrington (51)	2023	6609	163Weeks	63.3 (13.9)	4417	2192	CKD	Empagliflozin10 mg	Placebo
Cefalu (52)	2015	922	104Weeks	62.9 (7.32)	624	298	T2DMwith CVD	Dapagliflozin10 mg	Placebo
Zinman (12)	2014	7020	220Weeks	63.1 (8.6)	5016	2004	T2DMwith CVD	Empagliflozin10/25 mg	Placebo
Neal (53)	2013	4327	338Weeks	62.4 (8.02)	2861	1466	T2DMwith CVD	Canagliflozin100/300mg	Placebo
Leiter (54)	2013	965	104Weeks	63.8 (7.31)	644	321	T2DMwith CVD	Dapagliflozin10 mg	Placebo
Cannon (55)	2018	8238	288Weeks	64.4 (8.1)	5769	2469	T2DMwith CVD	Ertugliflozin5/15 mg	Placebo
Wiviott (56)	2018	17143	269Weeks	63.9 (6.8)	7907	9236	T2DMwith CVD	Dapagliflozin10 mg	Placebo
Szarek (57)	2021	1216	95Weeks	68.9 (9.1)	810	406	T2DM with HF	Sotagliflozin200/400 mg	Placebo
Wason (58)	2021	787	52Weeks	69.5 (7.9)	444	343	T2DM with CKD	Sotagliflozin200/400 mg	Placebo
Grunberger (59)	2018	467	67Weeks	67.3 (8.6)	231	236	T2DM with CKD	Ertugliflozin5/10/15 mg	Placebo
Allegretti (60)	2021	312	24Weeks	69.6 (8.32)	196	116	T2DM with CKD	Bexagliflozin20 mg	Placebo
Cherney (61)	2021	277	52Weeks	67.4 (9.3)	135	142	T2DM with CKD	Sotagliflozin200/400 mg	Placebo
Barnett (62)	2014	738	52Weeks	63.9 (8.8)	430	308	T2DM with CKD	Empagliflozin10/25 mg	Placebo
Jardine (14)	2017	4397	264Weeks	63 (9.2)	290	4107	T2DM with CKD	Canagliflozin100 mg	Placebo
Neal (63)	2017	5807	154Weeks	64 (8.35)	3648	2159	T2DM with CKD+CVD	Canagliflozin100/300mg	Placebo
Bhatt (64)	2021	10577	118Weeks	68.3 (8.4)	5830	4747	T2DM with HF+CKD	Sotagliflozin200/400 mg	Placebo

T2DM, type 2 diabetes mellitus; HFrEF, heart failure with reduced ejection fraction; HFpEF, heart failure with preserved ejection fraction; HF, heart failure; CVD, cardiovascular disease; CKD, chronic kidney disease.

### 3.2 Risk of bias assessment

The overall risk of bias across the included studies was low, with most studies demonstrating high or average quality. Specifically, 28 RCTs reported adequate random sequence generation, minimizing the risk of selection bias. However, eight studies had an unclear risk of hidden bias, highlighting the need for further clarification regarding their randomization methods. Only one studies demonstrated an unclear risk of bias related to blinding, suggesting that the risk of outcome bias was extremely low. Two studies exhibited a risk of bias due to incomplete data, and four studies had an unclear risk regarding selective reporting. Additionally, four studies had an unclear risk of other biases, which might impact the results.

**Figure 2:**
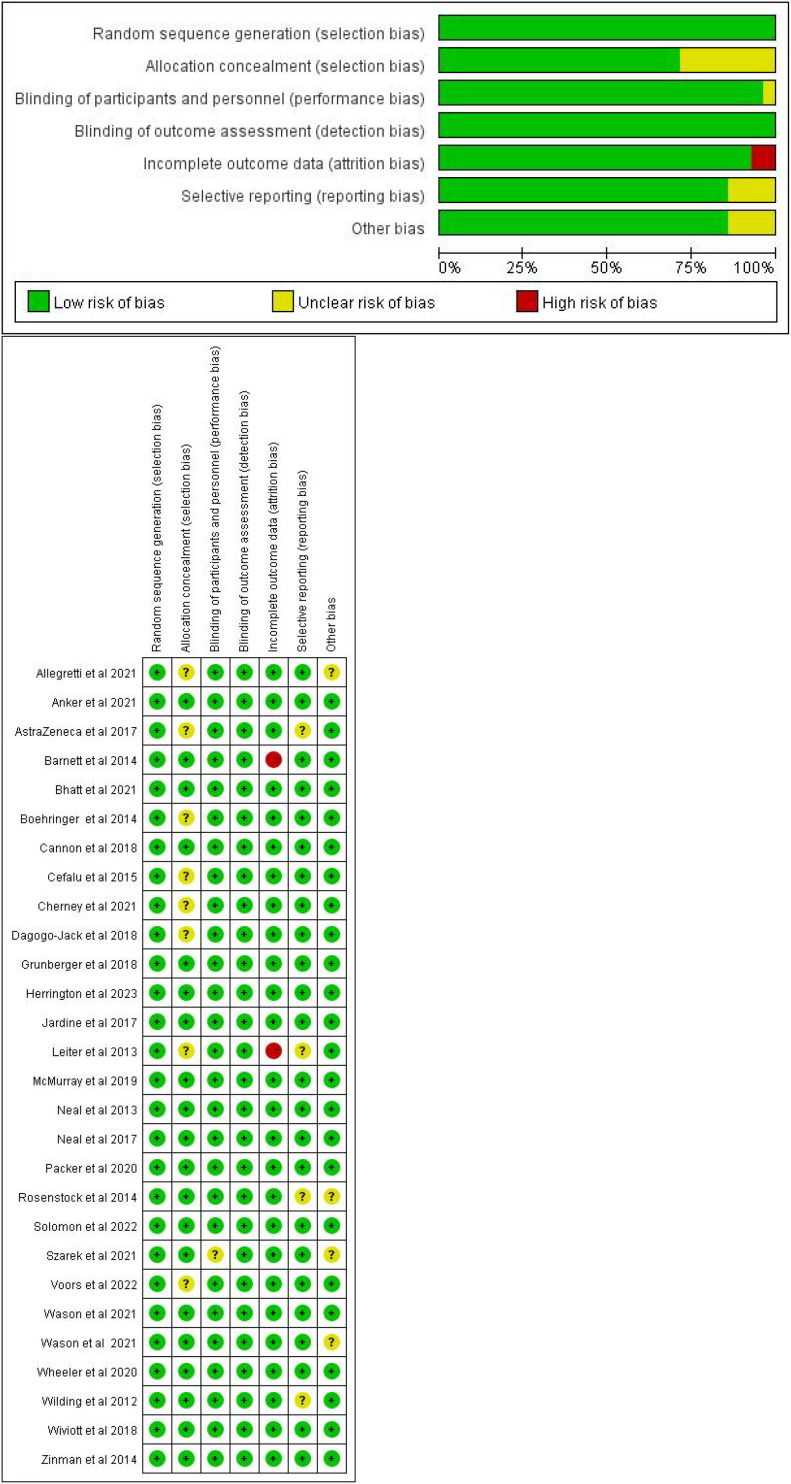
Risk of bias assessment for each trial.

Overall, while the studies generally show robust quality, there are some uncertainties regarding hidden biases, data loss, selective reporting, and other potential biases that should be considered when interpreting the findings (**Figure 2**).

### 3.3 Risk of cancer in the population in general

**Figure 3:**
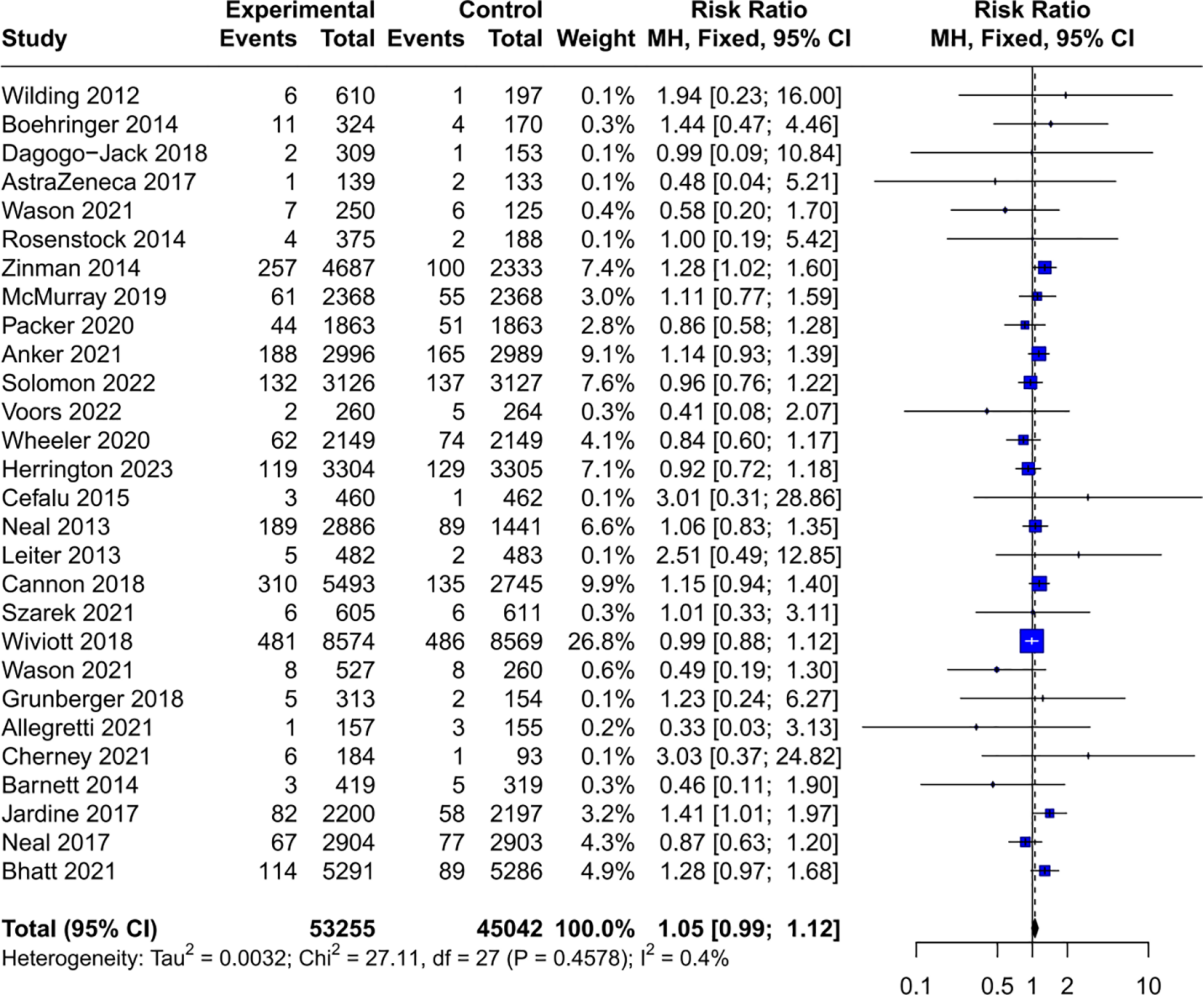
Forest plot of overall cancer risk. SGLT2, sodium-glucose cotransporter 2; CI, confidence interval.

Using a fixed-effects model, the meta-analysis revealed no significant increase in overall cancer risk associated with SGLT2 inhibitors compared to control groups (RR = 1.05, 95% CI [0.99, 1.12]), with low heterogeneity (*I*²= 0.4%, *P* = 0.46), indicating that the effect of SGLT2 inhibitors on cancer risk was consistent across the included trials (**Figure 3**). The low I² value supports the appropriateness of using a fixed-effect model, as it suggests that the true effect size was similar across studies.

For common cancer types, subgroup analyzes found no increase in the risk of digestive system cancers (RR = 1.10, 95% CI [0.97, 1.25]) (**Supplementary Figure 1**), respiratory system cancers (RR = 1.02, 95% CI [0.85, 1.22]) **Supplementary Figure 2**), squamous cell carcinoma (RR = 0.87, 95% CI [0.68, 1.11]) (**Supplementary Figure 3**), breast cancer (RR = 0.97, 95% CI [0.74, 1.27]) (**Supplementary Figure 4**), prostate cancer (RR = 0.88, 95% CI [0.72, 1.07]) (**Supplementary Figure 5**), and bladder cancer (RR = 0.90, 95% CI [0.67, 1.21]) (**Supplementary Figure 6**).

### 3.4 Subgroups analysis

**Figure 4:**
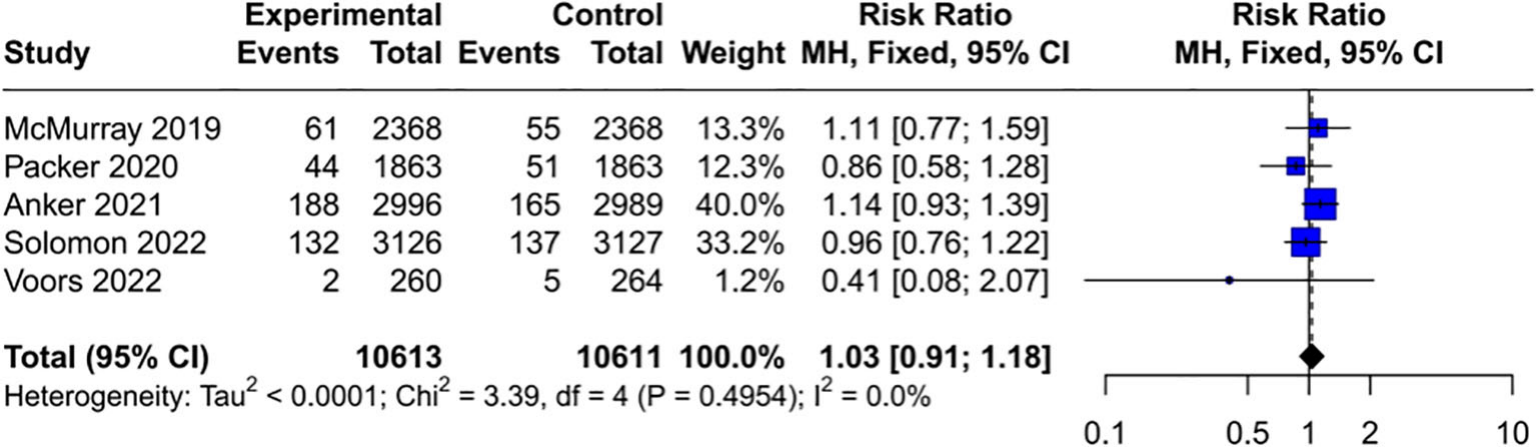
Forest plot of cancer risk in HF. HF, heart failure.

**Figure 5:**
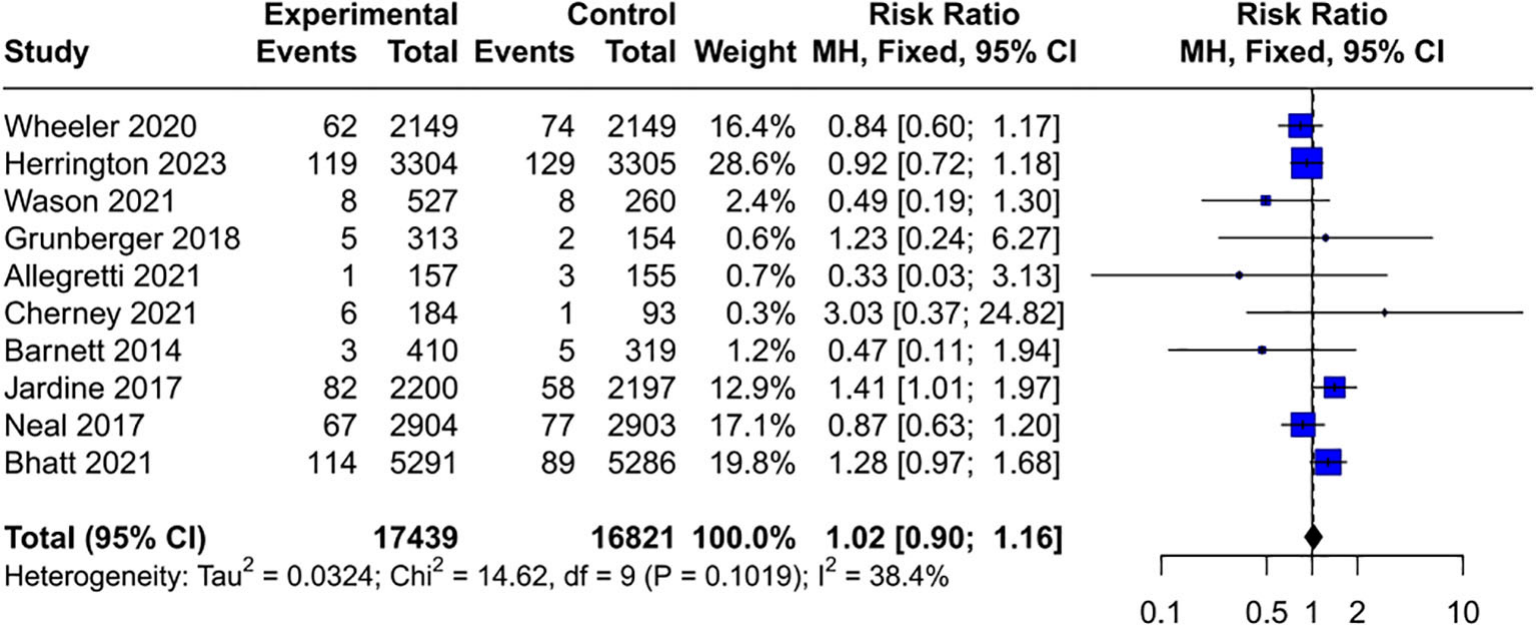
Forest plot of cancer risk in CKD. CKD, chronic kidney disease.

**Figure 6:**
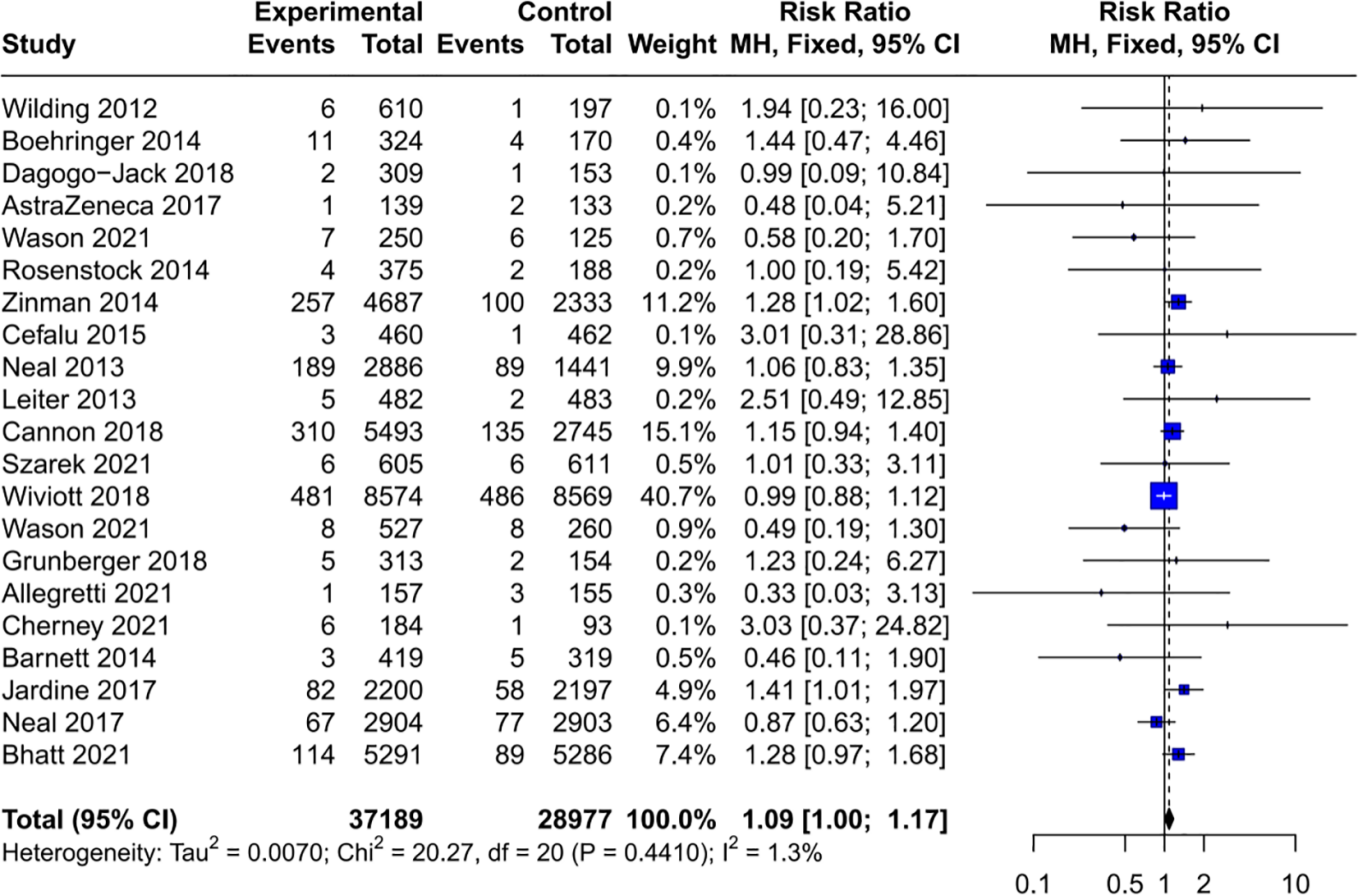
Forest plot of cancer risk in T2DM. T2DM, type 2 diabetes mellitus.

Given that baseline disease status may influence cancer susceptibility, the cancer risk associated with SGLT2 inhibitors was assessed in various subgroups. The results indicated that SGLT2 inhibitors do not increase the risk of cancer in people with HF (RR = 1.03, 95% CI [0.91, 1.18]) (**Figure 4**) and CKD (RR = 1.02, 95% CI [0.90, 1.16]) (**Figure 5**). Interestingly, the results of this study cannot completely rule out the possibility that SGLT2 inhibitors may increase the cancer risk in T2DM (RR = 1.09, 95% CI [1.00, 1.17]) (**Figure 6**), with no heterogeneity (*I*²= 1.3%, *P* = 0.44). In patients with T2DM and comorbidities such as cardiovascular disease (CVD) (RR = 1.08, 95% CI [0.99, 1.18]) (**Supplementary Figure 7**) or CKD (RR = 1.20, 95% CI [0.90, 1.61]) (**Supplementary Figure 8**), no significant increase in cancer incidence was observed.

Subgroup analysis based on the type of SGLT2 inhibitor revealed that SGLT2 inhibitors (Ertugliflozin, Dapagliflozin, Empagliflozin, Canagliflozin, Sotagliflozin) did not show any significant association with increased cancer risk (**Supplementary Figures S9**–**S13**).

### 3.5 Publication bias

**Figure 7:**
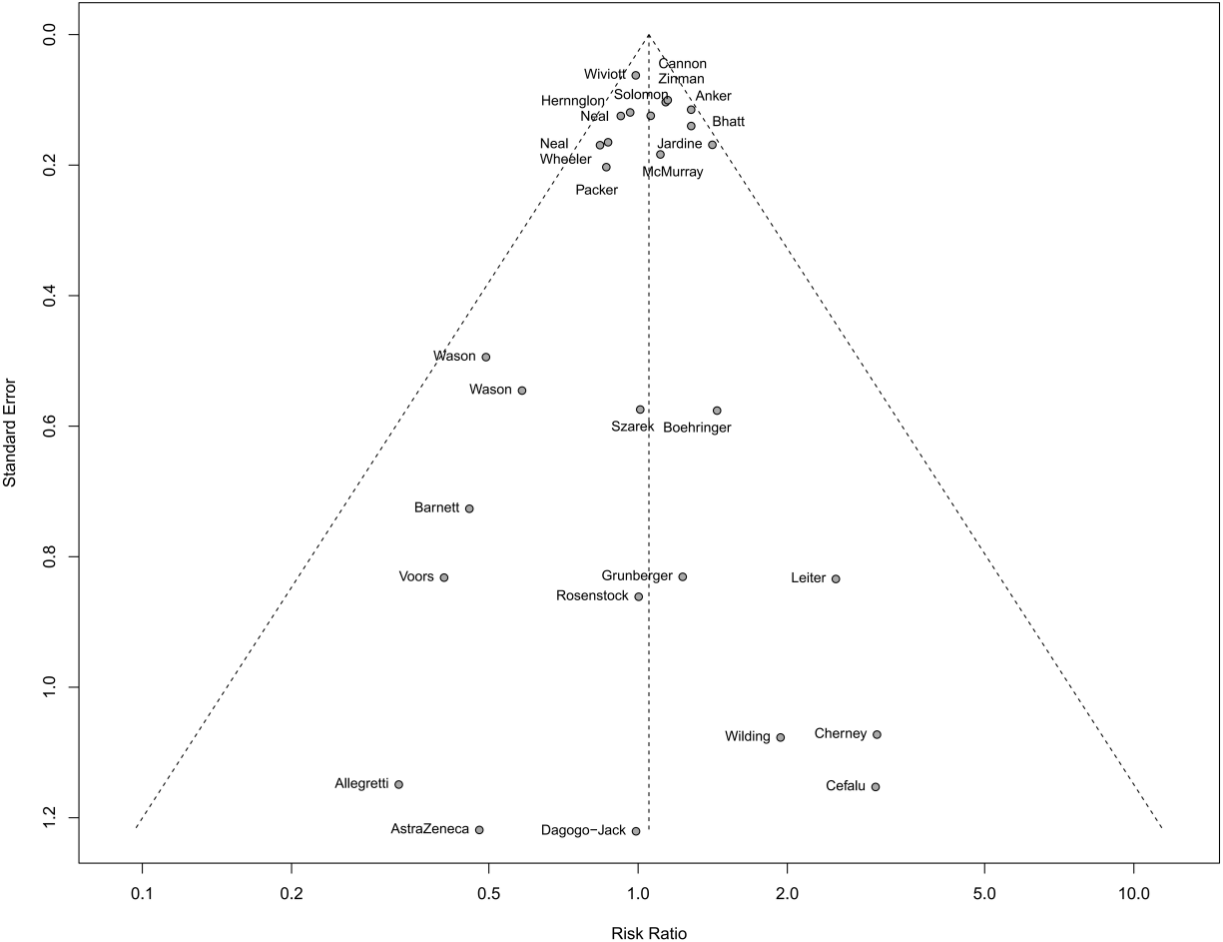
Funnel plot of the association between SGLT2 inhibitors and cancer risk.

To evaluate the potential for publication bias, funnel plots were constructed, and Begg’s test was performed. The funnel plot showed no significant asymmetry, indicating no substantial publication bias (**Figure 7**). Begg’s and Egger’s test confirmed this result with a p-value of 0.9842 and 0.6445, suggesting that publication bias did not significantly influence the findings.

## 4 Discussion

This meta-analysis included diverse populations, such as patients with T2DM, CKD, HF, and T2DM-related complications, focusing on six FDA-approved SGLT2 inhibitors. A primary concern with the long-term use of SGLT2 inhibitors is the potential risk of cancer. Elevated blood glucose can significantly increase cancer risk and impair chemotherapy responses by promoting cell growth and resistance mechanisms (65–69). In addition to influencing blood glucose levels, preclinical studies have also found that SGLT2 inhibitors can reverse hyperinsulinemia (70). Interestingly, this mechanism does not involve direct inhibition of SGLT2 in tumor cells. This suggests that the impact of SGLT2 inhibitors on cancer cells is more related to their systemic metabolic effects rather than direct action on tumor cell glucose uptake. Evidence from clinical trials remains inconclusive. Some studies suggest SGLT2 inhibitors, particularly Canagliflozin, may increase the risk of breast and bladder cancer (53, 71), with The CANVAS trial showing a slightly higher risk of breast cancer, although not statistically significant (63). In contrast, the EMPA-REG OUTCOME trial found no increased cancer risk in patients treated with Empagliflozin (12). These findings highlight the need for further investigation into the long-term safety of SGLT2 inhibitors and their potential effects on tumor growth.

Our analysis, based on 98,297 patients, revealed no significant overall cancer risk from SGLT2 inhibitors, consistent with previous analyzes (72, 73). Common types of SGLT2 inhibitors do not increase the overall risk of cancer either. Furthermore, in an analysis of several common cancers included in the studies, we found that the use of SGLT2 inhibitors did not increase the risk of bladder, breast, prostate, squamous, respiratory, and digestive cancers. Multiple studies have shown that SGLT2 inhibitors do not increase the risk of breast cancer and can reduce the mortality rate of breast cancer (74, 75). This is consistent with the results of this study. At present, there is still a lack of actual clinical evidence regarding the risk of bladder cancer. Therefore, this study analyzed bladder cancer. Consistent with previous analysis results, SGLT2 inhibitors can reduce the risk of bladder cancer, but there is no statistical significance (75). Although the results were not statistically significant, the findings of this study suggest that SGLT2 inhibitors may reduce the risk of squamous cell carcinoma and prostate cancer in the studied populations. Previous research has shown a positive correlation between blood glucose levels and the risk of head and neck squamous cell carcinoma (76), and the growth of squamous cell carcinoma can be inhibited through a ketogenic diet combined with the use of SGLT2 inhibitors (76). Additionally, recent studies have demonstrated that hyperglycemia can continuously promote the expression of specific genes, thereby creating a favorable microenvironment for the initiation and progression of prostate cancer (77, 78). Clinical investigations have also indicated that blood glucose control may influence prostate health in diabetic patients (79–81). However, the current study has several limitations, including the homogeneity of the study sample and the relatively short duration of follow-up. Therefore, future large-scale and long-term RCTs are needed to further assess the impact of SGLT2 inhibitors on cancer risk across diverse populations, particularly those with varying disease backgrounds, to provide more robust evidence for clinical treatment strategies.

Moreover, the analysis results indicate that the use of SGLT2 inhibitors may slightly increase the cancer risk of T2DM. The analysis included a total of 98,297 patients, of which 66,166 (67.3%) had a diagnosis of T2DM, while 32,121 (32.7%) did not. Within the T2DM group, 2,973 patients (4.5%) had no comorbidities, while the remaining 63,193 patients (95.5%) had one or more comorbid conditions. This distribution may have contributed to the variation in cancer risk across studies. Among the T2DM subgroup, a total of 2,646 patients were diagnosed with cancer, with 1,568 in the SGLT2 inhibitor group and 1,078 in the placebo group. The absolute increase in cancer diagnoses in the SGLT2 inhibitor group relative to the placebo group was 490 cases, and the potential number to harm, calculated as the excess cancer diagnoses attributable to SGLT2 inhibitor use, was approximately 179 cases. However, this increase, while notable, does not necessarily imply causality.

Upon examining the studies included in analysis, we identified that the data included from five studies significantly influenced RR. The baseline characteristics of the populations included in Cefalu2015 (52) and Leiter2013 (44) showed that approximately 42% to 45% of participants in both the SGLT2 inhibitor and placebo groups were elderly (≥65 years). However, the large-scale study by Wiviott2018, in which approximately 46% of participants in both the SGLT2 inhibitor and placebo groups were aged ≥65, found no increased cancer risk associated with the use of SGLT2 inhibitors in the T2DM population. Additionally, the sample size in Cefalu2015 was insufficient to draw conclusions regarding rare events (52, 82). These findings underscore the importance of recognizing that minor imbalances in small-scale studies may not accurately reflect the true cancer risk. The study by Cherney2021 (61) may lack clinical significance due to the relatively small sample size, and Cherney2021 had a relatively short duration of 52 weeks. Short-term studies may not fully capture the delayed effects of drug use, including the development of cancer, which often takes years to manifest. Furthermore, statistical analyzes conducted by Zinman2014 (12) and Jardine2017 (14) indicated that the use of SGLT2 inhibitors was associated with an increased overall risk of cancer, with the findings reaching statistical significance. A closer examination of the statistical results from Zinman2014 revealed that adverse events accounted for approximately 90% of incidents in both the placebo and SGLT2 inhibitor groups. As this meta-analysis categorized different doses of SGLT2 inhibitors into one experimental group, the number of cancer patients observed was significantly higher. It is also important to note that the participants in the Jardine2017 study had type 2 diabetes accompanied by chronic kidney disease with proteinuria, conditions more severe than those in other included studies. As a result, it may lead to a higher baseline cancer risk in the study population, making it difficult to attribute the observed cancer risk specifically to the use of SGLT2 inhibitors (83–85). Furthermore, considering the confidence interval was 1.00, the result did not reach statistical significance, which indicates that although there is a slight association between SGLT2 inhibitors and the cancer risk of T2DM patients, the evidence does not support a causal relationship. The observed increase in the rate of cancer diagnosis may be influenced by research-specific factors, such as small sample size, baseline characteristics, and reporting bias. Therefore, it cannot be confirmed at present that the use of SGLT2 inhibitors is related to the cancer risk of patients with T2DM.

In summary, this study affirms the safety of SGLT2 inhibitors in HF, T2DM, CKD and T2DM related comorbidities. Furthermore, no significant increase in cancer risk was observed with any of the six SGLT2 inhibitors included in this study. These findings provide important evidence supporting the safety of these medications for patients with long-term underlying conditions and may enhance both patient and clinician confidence in the use of these drugs. The reliability of the safety data further supports the broad application of SGLT2 inhibitors as treatment options for various chronic diseases, thereby offering patients effective therapeutic alternatives and improving their quality of life. However, considering the potential upward trend in cancer risk observed and the limitations of the current analysis, further long-term RCTs are needed to evaluate the long-term relationship between SGLT2 inhibitors and cancer risk. Additionally, this meta-analysis does not include data on cancer subtypes in patients with T2DM. Future research should also focus on investigating the underlying biological mechanisms contributing to the potential cancer risk, to better understand the observed association.

## 5 Conclusions

Data from RCTs indicate that SGLT2 inhibitors do not increase overall cancer risk. Subgroup analysis showed that the use of SGLT2 inhibitors was temporarily safe for patients with different underlying diseases. Despite persistent concerns about potential cancer associations, current evidence does not support that SGLT2 inhibitors significantly increase the risk of cancer.

## Data Availability

Publicly available datasets were analyzed in this study. This data can be found here: ClinicalTrials.gov.
