# Supplementary figures and images for: Safety evaluation of Sodium-glucose cotransporter 2 inhibitors for cancer risk in specific populations: systematic review and meta-analysis
Source: Front Clin Diabetes Healthc. 2026 May 8;7:1775359. doi: 10.3389/fcdhc.2026.1775359 (PMC13193846; doi:10.3389/fcdhc.2026.1775359)

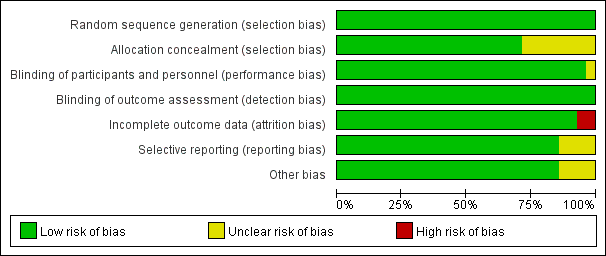

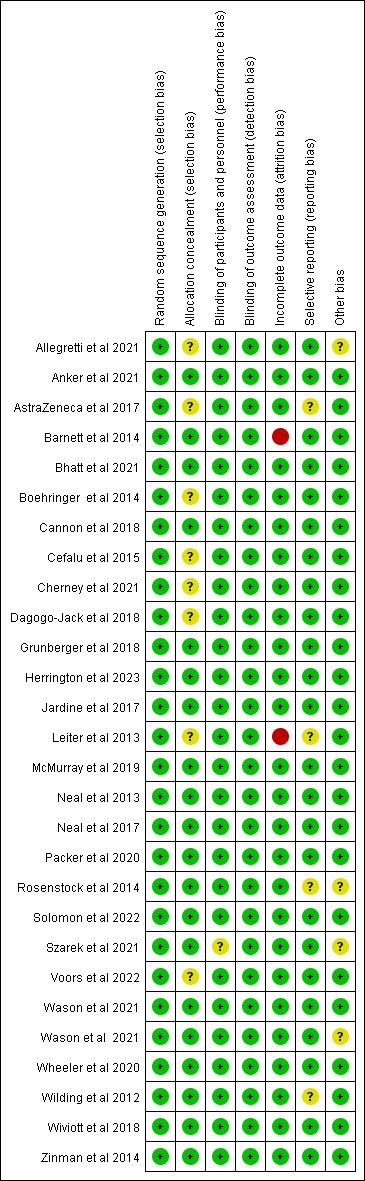

Supplement: Supplementary file 1 [file Table1.docx]
