# Supplementary material for: Safety evaluation of Sodium-glucose cotransporter 2 inhibitors for cancer risk in specific populations: systematic review and meta-analysis
Source: Front Clin Diabetes Healthc. 2026 May 8;7:1775359. doi: 10.3389/fcdhc.2026.1775359 (PMC13193846; doi:10.3389/fcdhc.2026.1775359)
Supplement: Supplementary file 2 [file Table2.docx]

**Identification of studies via databases and registers**

Records removed *before screening*:

Duplicate records removed (n = 4732)

Records marked as ineligible by automation tools (n = 0)

Records removed for other reasons (n = 0)

Records identified from*:

Databases (n = 8533):

PubMed (n= 1995)

CENTRAL (n= 3199)

Web of science (n = 2779)

ClinicalTrials.gov (n = 560)

Registers (n = 0)

**Identification**

Records screened

(n = 3801)

Records excluded**

(n = 2420)

Reports sought for retrieval

(n = 1381)

Reports not retrieved

(n = 394)

**Screening**

Reports assessed for eligibility

(n = 987)

Reports excluded:

Secondary report of excluded study (n=250)

Trials with trial duration less than 12 weeks (n = 9)

Non-RCT (n = 14)

No report of cancer or the number of cancers reported is too low (n=383)

Ongoing/Recruiting (n=289)

control group treatment is not a placebo (n=14)

Studies included in review

(n = 28)

**Included**

*Consider, if feasible to do so, reporting the number of records identified from each database or register searched (rather than the total number across all databases/registers).

**If automation tools were used, indicate how many records were excluded by a human and how many were excluded by automation tools.

Source: Page MJ, et al. BMJ 2021;372:n71. doi: 10.1136/bmj.n71.

This work is licensed under CC BY 4.0. To view a copy of this license, visit <https://creativecommons.org/licenses/by/4.0/>
