# Supplementary material for: Safety evaluation of Sodium-glucose cotransporter 2 inhibitors for cancer risk in specific populations: systematic review and meta-analysis
Source: Front Clin Diabetes Healthc. 2026 May 8;7:1775359. doi: 10.3389/fcdhc.2026.1775359 (PMC13193846; doi:10.3389/fcdhc.2026.1775359)
Supplement: Supplementary file 4 [file Table4.docx]

| Data source | Search strategy | Results | Search date |
| --- | --- | --- | --- |
| PubMed | (((((((((((((Sodium glucose co-transporter) OR (SGLT2 inhibitors OR SGLT-2 inhibitors OR SGLT 2 inhibitors)) OR (Tofogliflozin OR Apleway OR Deberza OR CSG452)) OR (Empagliflozin OR Jardiance)) OR (Dapagliflozin OR Farxiga OR Forxiga)) OR (Canagliflozin OR Invokana)) OR (Sotagliflozin OR LX4211)) OR (Luseogliflozin OR Lusefi)) OR (Ipragliflozin OR Suglat)) OR (Remogliflozin OR BHV091009)) OR (Sergliflozin OR GW869682X)) OR (Ertugliflozin OR MK-8835 OR PF-04971729)) AND (placebo)) AND ((((randomized controlled trial[Publication Type]) OR (randomized[Title/Abstract])) OR (placebo[Title/Abstract])) OR ((RCT) OR (RCTs))) | 1995 | 4/16/2024 |
| CENTRAL | Sodium glucose co-transporter OR SGLT2 inhibitors OR SGLT-2 inhibitors OR SGLT 2 inhibitors OR Tofogliflozin OR Empagliflozin OR Dapagliflozin OR Canagliflozin OR Sotagliflozin OR Luseogliflozin OR Ipragliflozin OR Remogliflozin OR Sergliflozin OR Ertugliflozin AND placebo AND (randomized controlled trial OR randomized OR placebo OR RCT OR RCTs):ab,ti,kw | 3199 | 4/16/2024 |
| Web of science | TS=(Sodium glucose co-transporter OR SGLT2 inhibitors OR SGLT-2 inhibitors OR SGLT 2 inhibitors OR Tofogliflozin OR Empagliflozin OR Dapagliflozin OR Canagliflozin OR Sotagliflozin OR Luseogliflozin OR Ipragliflozin OR Remogliflozin OR Sergliflozin OR Ertugliflozin) AND TS=(placebo) AND TS=(randomized controlled trial OR randomized OR placebo OR RCT OR RCTs) | 2779 | 4/16/2024 |
| ClinicalTrials.gov | (Tofogliflozin OR Empagliflozin OR Dapagliflozin OR Canagliflozin OR Sotagliflozin OR Luseogliflozin OR Ipragliflozin OR Remogliflozin OR Sergliflozin OR Ertugliflozin) AND placebo | 560 | 4/16/2024 |

**Table 1** Search Strategy. SGLT2, Sodium-glucose cotransporter 2; RCT, randomized controlled trial.


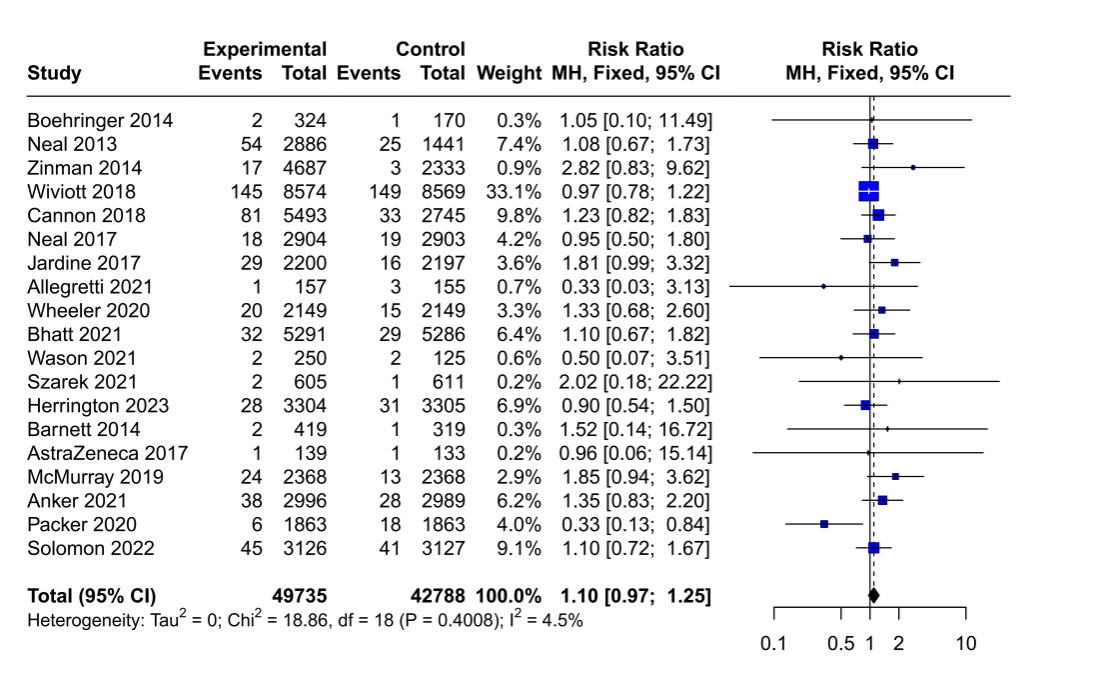


**Fig. S1** Forest plot of digestive system cancer risk.


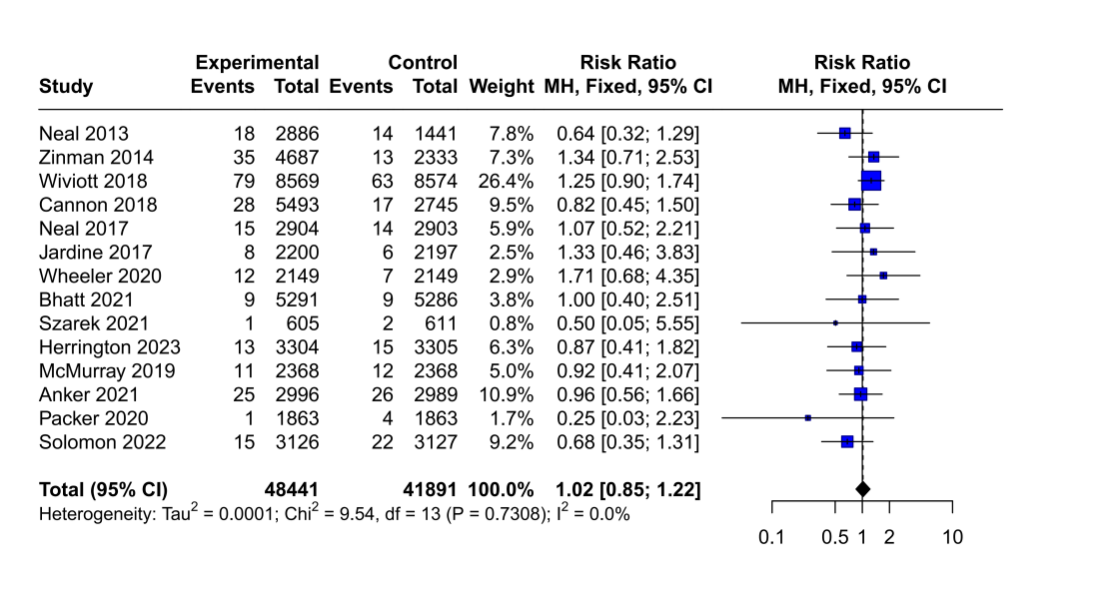


**Fig. S2** Forest plot of respiratory system cancer risk.


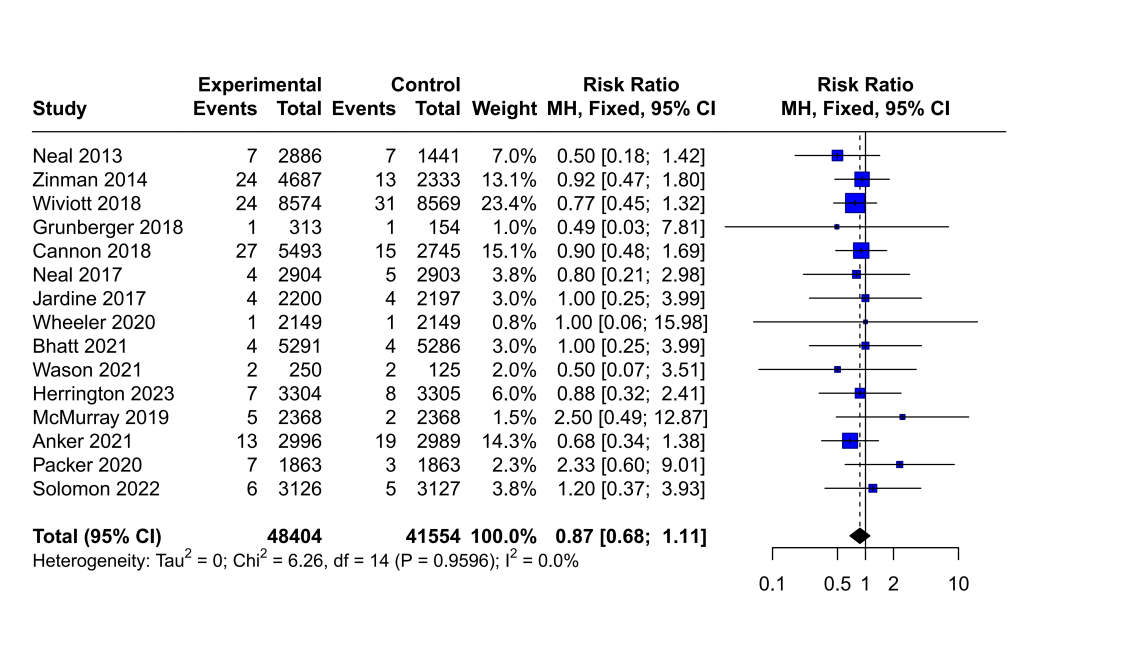


**Fig. S3** Forest plot of squamous cell cancer risk.


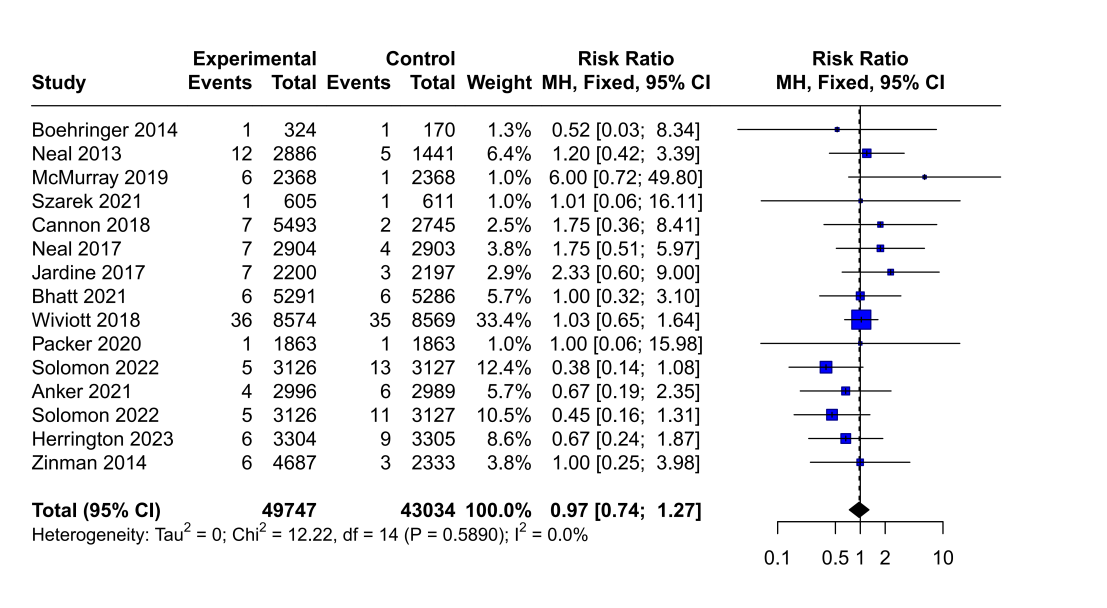


**Fig. S4** Forest plot of breast cancer risk.


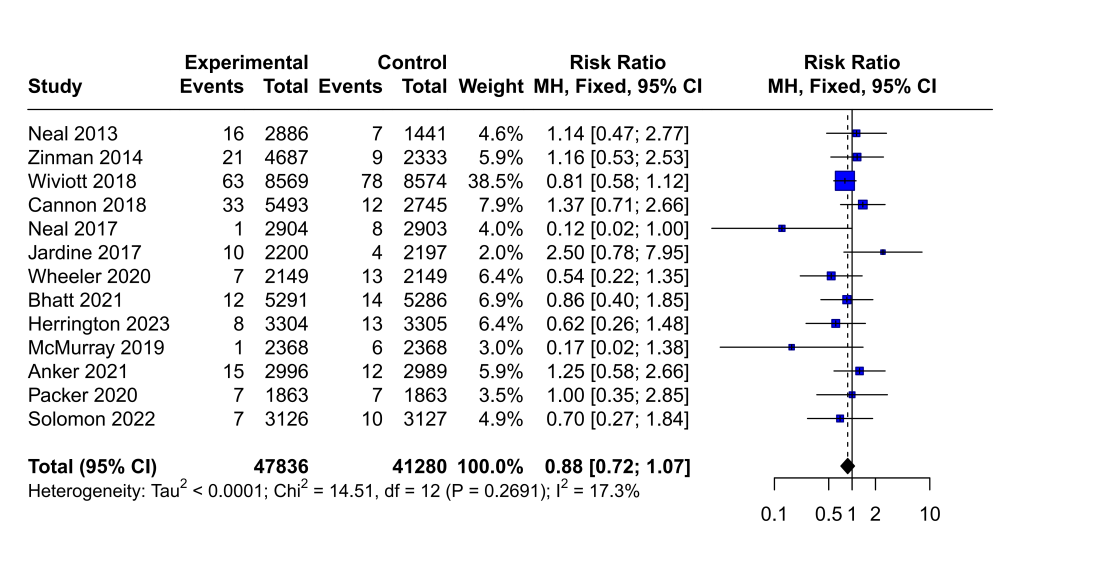


**Fig. S5** Forest plot of prostatic cancer risk.


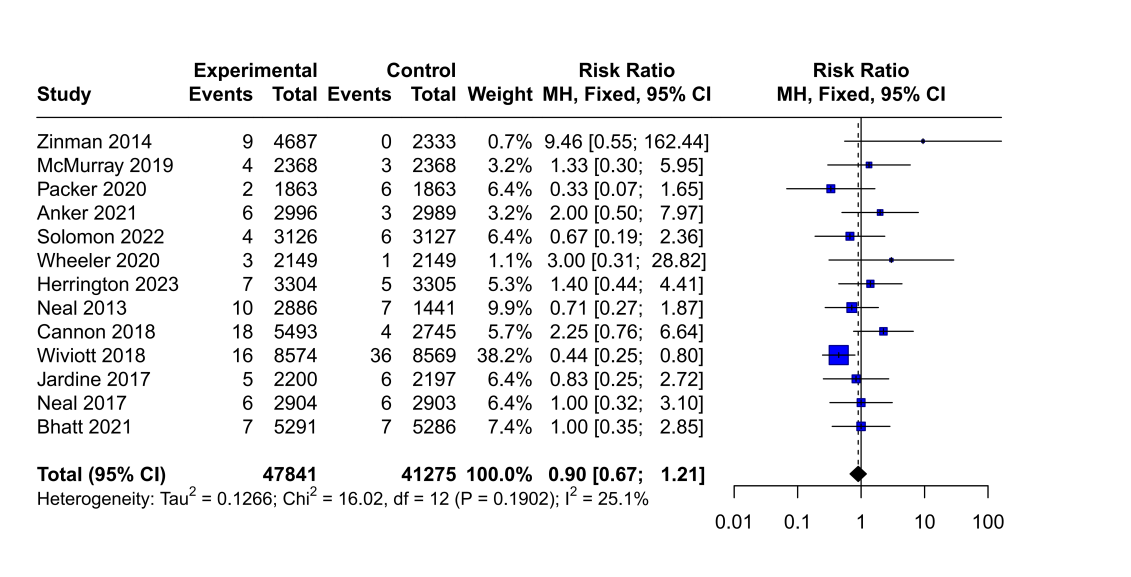


**Fig. S6**Forest plot of bladder cancer risk.


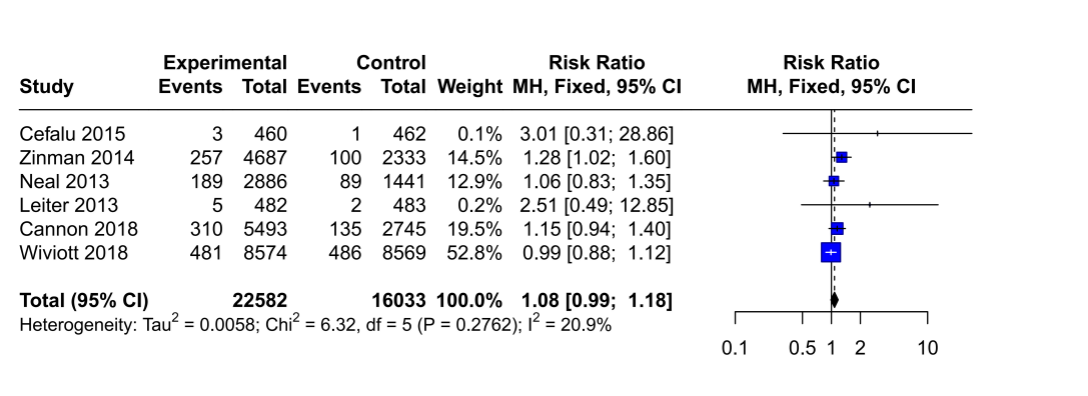


**Fig. S7** Forest plot of cancer risk in T2DM with CVD. T2DM, type 2 diabetes mellitus; CVD, cardiovascular disease.


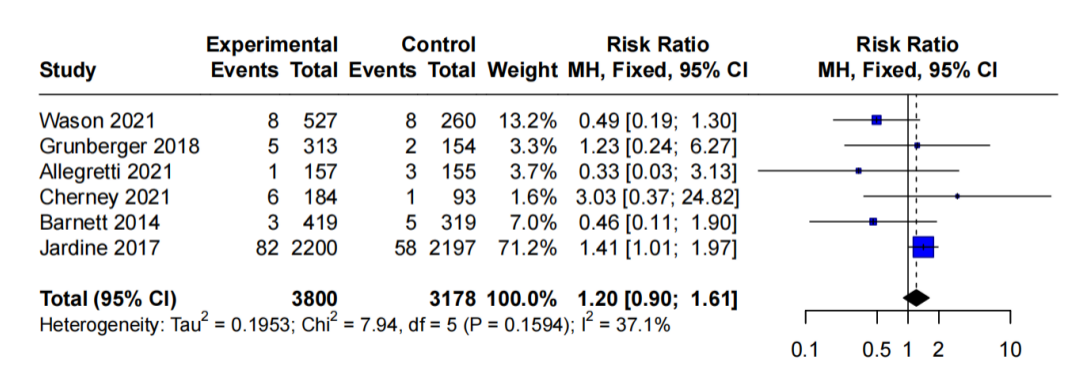


**Fig. S8** Forest plot of cancer risk in T2DM with CKD. T2DM, type 2 diabetes mellitus; CKD, chronic kidney disease.


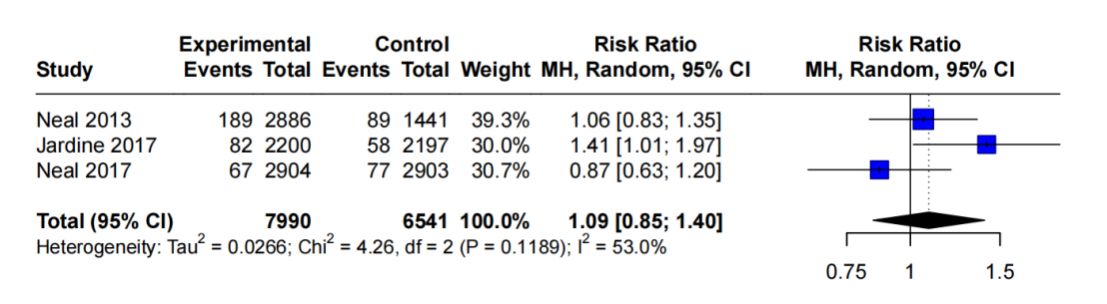


**Fig. S9** Forest plot of cancer risk in patients using Canagliflozin.


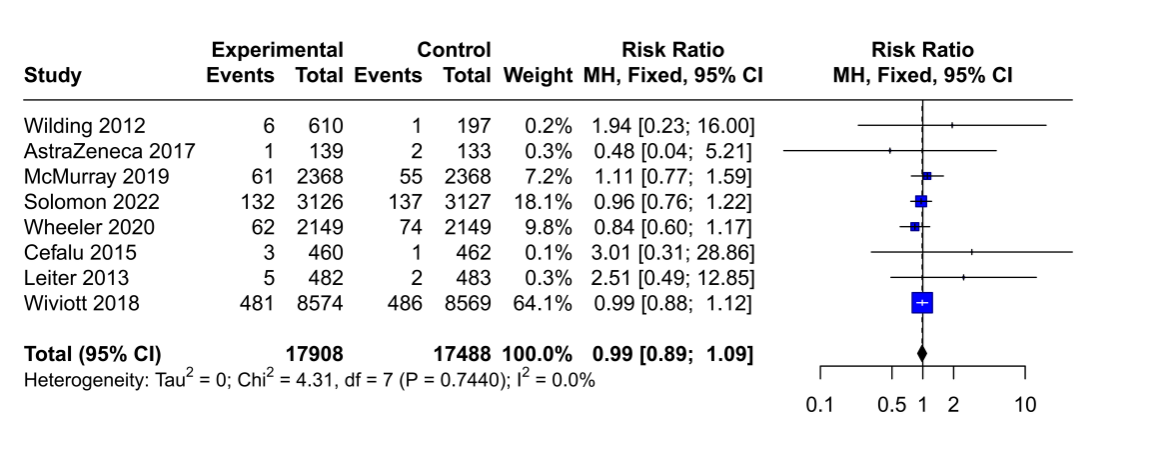


**Fig. S10** Forest plot of cancer risk in patients using Dapagliflozin.


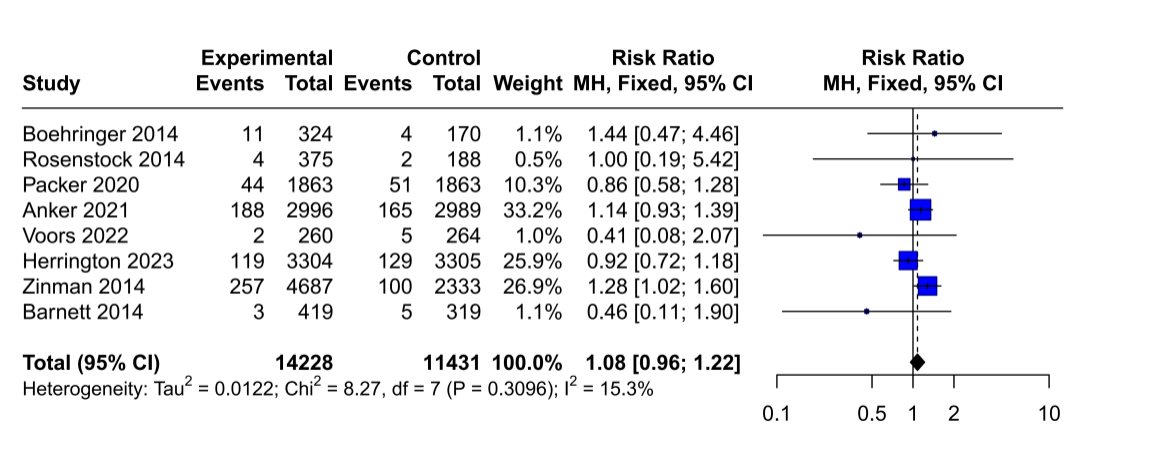


**Fig. S11** Forest plot of cancer risk in patients using Empagliflozin.


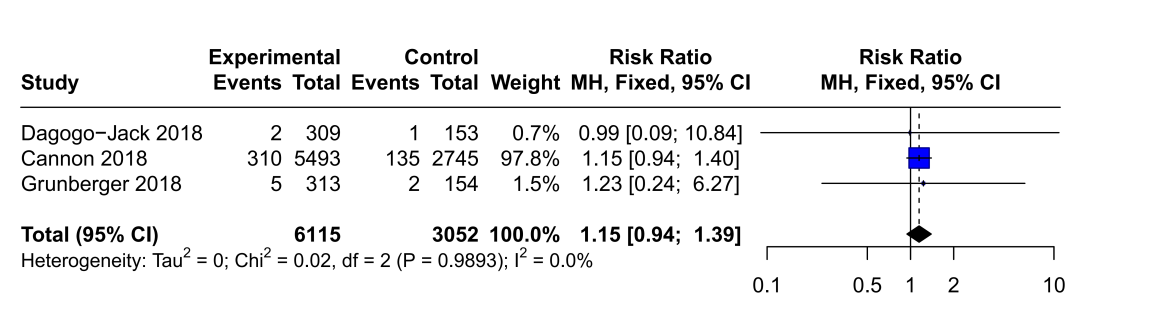


**Fig. S12** Forest plot of cancer risk in patients using Ertugliflozin.


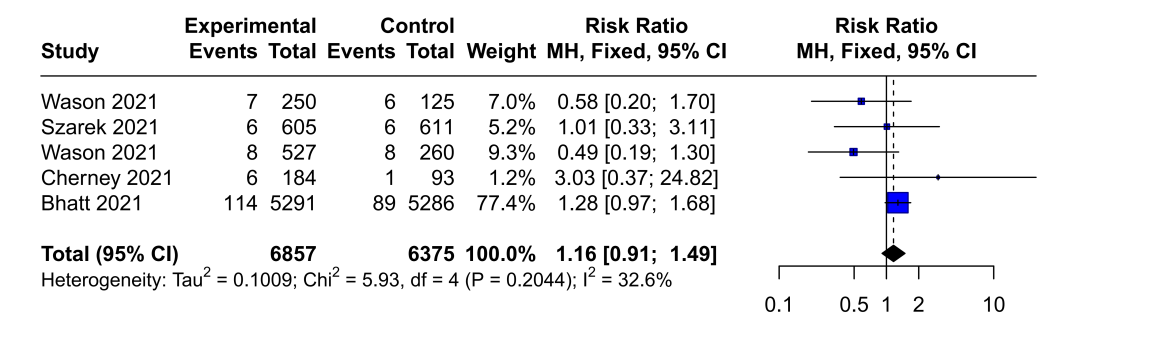


**Fig. S13** Forest plot of cancer risk in patients using Sotagliflozin.
