# Supplementary material for: Safety evaluation of Sodium-glucose cotransporter 2 inhibitors for cancer risk in specific populations: systematic review and meta-analysis
Source: Front Clin Diabetes Healthc. 2026 May 8;7:1775359. doi: 10.3389/fcdhc.2026.1775359 (PMC13193846; doi:10.3389/fcdhc.2026.1775359)
Supplement: Supplementary file 5 [file Table5.docx]

1. Search strategy

| Data source | Search strategy | Results | Search date |
| --- | --- | --- | --- |
| **PubMed** | (((((((((((((Sodium glucose co-transporter) OR (SGLT2 inhibitors OR SGLT-2 inhibitors OR SGLT 2 inhibitors)) OR (Tofogliflozin OR Apleway OR Deberza OR CSG452)) OR (Empagliflozin OR Jardiance)) OR (Dapagliflozin OR Farxiga OR Forxiga)) OR (Canagliflozin OR Invokana)) OR (Sotagliflozin OR LX4211)) OR (Luseogliflozin OR Lusefi)) OR (Ipragliflozin OR Suglat)) OR (Remogliflozin OR BHV091009)) OR (Sergliflozin OR GW869682X)) OR (Ertugliflozin OR MK-8835 OR PF-04971729)) AND (placebo)) AND ((((randomized controlled trial[Publication Type]) OR (randomized[Title/Abstract])) OR (placebo[Title/Abstract])) OR ((RCT) OR (RCTs))) | 1995 | 4/16/2024 |
| **CENTRAL** | Sodium glucose co-transporter OR SGLT2 inhibitors OR SGLT-2 inhibitors OR SGLT 2 inhibitors OR Tofogliflozin OR Empagliflozin OR Dapagliflozin OR Canagliflozin OR Sotagliflozin OR Luseogliflozin OR Ipragliflozin OR Remogliflozin OR Sergliflozin OR Ertugliflozin AND placebo AND (randomized controlled trial OR randomized OR placebo OR RCT OR RCTs):ab,ti,kw | 3199 | 4/16/2024 |
| **Web of science** | TS=(Sodium glucose co-transporter OR SGLT2 inhibitors OR SGLT-2 inhibitors OR SGLT 2 inhibitors OR Tofogliflozin OR Empagliflozin OR Dapagliflozin OR Canagliflozin OR Sotagliflozin OR Luseogliflozin OR Ipragliflozin OR Remogliflozin OR Sergliflozin OR Ertugliflozin) AND TS=(placebo) AND TS=(randomized controlled trial OR randomized OR placebo OR RCT OR RCTs) | 2779 | 4/16/2024 |
| **ClinicalTrials.gov** | (Tofogliflozin OR Empagliflozin OR Dapagliflozin OR Canagliflozin OR Sotagliflozin OR Luseogliflozin OR Ipragliflozin OR Remogliflozin OR Sergliflozin OR Ertugliflozin) AND placebo | 560 | 4/16/2024 |

1. Search terms

**Pubmed**

| **Query** | **Results** |
| --- | --- |
| #1 Sodium glucose co-transporter  #2 SGLT2 inhibitors OR SGLT-2 inhibitors OR SGLT 2 inhibitors  #3 Tofogliflozin OR Apleway OR Deberza OR CSG452  #4 Empagliflozin OR Jardiance  #5 Dapagliflozin OR Farxiga OR Forxiga  #6 Canagliflozin OR Invokana  #7 Sotagliflozin OR LX4211  #8 Luseogliflozin OR Lusefi  #9 Ipragliflozin OR Suglat  #10 Remogliflozin OR BHV091009  #11 Sergliflozin OR GW869682X  #12 Ertugliflozin OR MK-8835 OR PF-04971729  #13 OR#1- #12 | 6453  10964  172  3210  3094  1950  352  169  311  51  18  291  14868 |
| #14 ((randomized controlled trial[Publication Type]) OR (randomized[Title/Abstract])) OR (placebo[Title/Abstract])  #15  (RCT) OR (RCTs)  #16 #14 OR #15 | 1057108  83000  1078699 |
| #17 placebo | 273973 |
| #18 #13 AND #16 | 1995 |

**Central**

| **Query** | **Results** |
| --- | --- |
| #1 Sodium glucose co-transporter  #2 SGLT2 inhibitors OR SGLT-2 inhibitors OR SGLT 2 inhibitors  #3 Tofogliflozin  #4 Empagliflozin  #5 Dapagliflozin  #6 Canagliflozin  #7 Sotagliflozin  #8 Luseogliflozin  #9 Ipragliflozin  #10 Remogliflozin  #11 Sergliflozin  #12 Ertugliflozin  #13 OR#1- #12 | 837  1590  113  1962  2126  834  163  111  180  38  8  223  5861 |
| #14 placebo | 394356 |
| #15 (randomized controlled trial OR randomized OR placebo OR RCT OR RCTs):ab,ti,kw | 1289044 |
| #16 #13 AND #14 AND #15 | 3199 |

**Web of science**

| **Query** | **Results** |
| --- | --- |
| #1 TS=(Sodium glucose co-transporter OR SGLT2 inhibitors OR SGLT-2 inhibitors OR SGLT 2 inhibitors OR Tofogliflozin OR Empagliflozin OR Dapagliflozin OR Canagliflozin OR Sotagliflozin OR Luseogliflozin OR Ipragliflozin OR Remogliflozin OR Sergliflozin OR Ertugliflozin) | 23054 |
| #2 TS=(placebo) | 440262 |
| #3 TS=(randomized controlled trial OR randomized OR placebo OR RCT OR RCTs) | 1822334 |
| #4 #1 AND #2 AND #3 | 2779 |
