# Supplementary material for: Safety evaluation of Sodium-glucose cotransporter 2 inhibitors for cancer risk in specific populations: systematic review and meta-analysis
Source: Front Clin Diabetes Healthc. 2026 May 8;7:1775359. doi: 10.3389/fcdhc.2026.1775359 (PMC13193846; doi:10.3389/fcdhc.2026.1775359)
Supplement: Supplementary file 6 [file Table6.docx]

| **First Author** | **Year** | **Number of patients** | **Follow-up Time** | **Mean Age (Standard Deviation)** | **sex** | | **Target Population** | **Theraputic regimen** | |
| --- | --- | --- | --- | --- | --- | --- | --- | --- | --- |
|  |  |  |  |  | **Male** | **Female** |  | **Experiment** | **Control** |
| Wilding [35] | 2012 | 807 | 104Weeks | 59.3  (8.22) | 382 | 425 | T2DM | Dapagliflozin2.5/5/10 mg | Placebo |
| Boehringer [36] | 2014 | 494 | 78Weeks | 58.8  (9.9) | 276 | 218 | T2DM | Empagliflozin10/25 mg | Placebo |
| Dagogo-Jack [37] | 2018 | 462 | 52Weeks | 59.1  (9.0) | 263 | 199 | T2DM | Ertugliflozin5/10/15 mg | Placebo |
| AstraZeneca [38] | 2017 | 272 | 24Weeks | 57.5  (8.70) | 130 | 142 | T2DM | Dapagliflozin10 mg | Placebo |
| Wason [39] | 2021 | 375 | 110Weeks | 66.3  (6.5) | 209 | 166 | T2DM | Sotagliflozin200/400 mg | Placebo |
| Rosenstock [40] | 2014 | 563 | 52Weeks | 56.7  (9.5) | 256 | 307 | T2DM | Empagliflozin10/25 mg | Placebo |
| McMurray [41] | 2019 | 4736 | 111Weeks | 66.3  (10.9) | 3635 | 1101 | HFrEF | Dapagliflozin10 mg | Placebo |
| Packer [42] | 2020 | 3726 | 148Weeks | 66.8  (11.0) | 2837 | 889 | HFrEF | Empagliflozin10 mg | Placebo |
| Anker [43] | 2021 | 5985 | 200Weeks | 71.9  (9.4) | 3312 | 2673 | HFpEF | Empagliflozin10 mg | Placebo |
| Solomon [44] | 2022 | 6253 | 168Weeks | 71.7  (9.6) | 3516 | 2737 | HFpEF | Dapagliflozin10 mg | Placebo |
| Voors [45] | 2022 | 524 | 13Weeks | 68.5  (13.2) | 351 | 173 | Acute HF | Empagliflozin10 mg | Placebo |
| Wheeler [46] | 2020 | 4298 | 153Weeks | 61.8  (12.1) | 2879 | 1419 | CKD | Dapagliflozin10 mg | Placebo |
| Herrington [47] | 2023 | 6609 | 163Weeks | 63.3  (13.9) | 4417 | 2192 | CKD | Empagliflozin10 mg | Placebo |
| Cefalu [31] | 2015 | 922 | 104Weeks | 62.9  (7.32) | 624 | 298 | T2DMwith CVD | Dapagliflozin10 mg | Placebo |
| Zinman [34] | 2014 | 7020 | 220Weeks | 63.1  (8.6) | 5016 | 2004 | T2DMwith CVD | Empagliflozin10/25 mg | Placebo |
| Neal [19] | 2013 | 4327 | 338Weeks | 62.4  (8.02) | 2861 | 1466 | T2DMwith CVD | Canagliflozin100/300mg | Placebo |
| Leiter [32] | 2013 | 965 | 104Weeks | 63.8  (7.31) | 644 | 321 | T2DMwith CVD | Dapagliflozin10 mg | Placebo |
| Cannon [48] | 2018 | 8238 | 288Weeks | 64.4  (8.1) | 5769 | 2469 | T2DMwith CVD | Ertugliflozin5/15 mg | Placebo |
| Wiviott [49] | 2018 | 17143 | 269Weeks | 63.9  (6.8) | 7907 | 9236 | T2DMwith CVD | Dapagliflozin10 mg | Placebo |
| Szarek [50] | 2021 | 1216 | 95Weeks | 68.9  (9.1) | 810 | 406 | T2DM with HF | Sotagliflozin200/400 mg | Placebo |
| Wason [51] | 2021 | 787 | 52Weeks | 69.5  (7.9) | 444 | 343 | T2DM with CKD | Sotagliflozin200/400 mg | Placebo |
| Grunberger [52] | 2018 | 467 | 67Weeks | 67.3  (8.6) | 231 | 236 | T2DM with CKD | Ertugliflozin5/10/15 mg | Placebo |
| Allegretti [53] | 2021 | 312 | 24Weeks | 69.6  (8.32) | 196 | 116 | T2DM with CKD | Bexagliflozin20 mg | Placebo |
| Cherney [33] | 2021 | 277 | 52Weeks | 67.4  (9.3) | 135 | 142 | T2DM with CKD | Sotagliflozin200/400 mg | Placebo |
| Barnett [54] | 2014 | 738 | 52Weeks | 63.9  (8.8) | 430 | 308 | T2DM with CKD | Empagliflozin10/25 mg | Placebo |
| Jardine [5] | 2017 | 4397 | 264Weeks | 63  (9.2) | 290 | 4107 | T2DM with CKD | Canagliflozin100 mg | Placebo |
| Neal [21] | 2017 | 5807 | 154Weeks | 64  (8.35) | 3648 | 2159 | T2DM with CKD+CVD | Canagliflozin100/300mg | Placebo |
| Bhatt [55] | 2021 | 10577 | 118Weeks | 68.3  (8.4) | 5830 | 4747 | T2DM with HF+CKD | Sotagliflozin200/400 mg | Placebo |

**Table 1** Overall baseline characteristics of the studies included in the meta analysis. T2DM, type 2 diabetes mellitus; HFrEF, heart failure with reduced ejection fraction; HFpEF, heart failure with preserved ejection fraction; HF, heart failure; CVD, cardiovascular disease; CKD, chronic kidney disease.
